# Addressing the gaps: sex differences in osteoarthritis of the knee

**DOI:** 10.1186/2042-6410-4-4

**Published:** 2013-02-04

**Authors:** Barbara D Boyan, Laura L Tosi, Richard D Coutts, Roger M Enoka, David A Hart, Daniel P Nicolella, Karen J Berkley, Kathleen A Sluka, C Kent Kwoh, Mary I O’Connor, Wendy M Kohrt, Eileen Resnick

**Affiliations:** 1 ISIS Research Network on Musculoskeletal Health, Society for Women’s Health Research, Washington, DC, USA; 2 Wallace H. Coulter Department of Biomedical Engineering, Georgia Institute of Technology, 315 Ferst Drive NW, Atlanta, GA, 30332-0363, USA; 3 Program in Neuroscience, Florida State University, Tallahassee, FL, 32306-4301, USA; 4 Mayo Clinic, 4500 San Pablo Road, Jacksonville, FL, USA; 5 Division of Orthopaedic Surgery and Sports Medicine, Children's National Medical Center, Washington, DC, 20010

**Keywords:** Sex differences in response to sex steroids, Knee biomechanics and osteoarthritis, Pain perception in knee osteoarthritis, Musculoskeletal tissues, Estrogen, Testosterone, Rapid actions, Ligaments, Tendons, Bones, Animal models of osteoarthritis, Knee as an organ

## Abstract

An introduction to the accompanying three papers.

## Review

### Introduction

**Figure 1:**
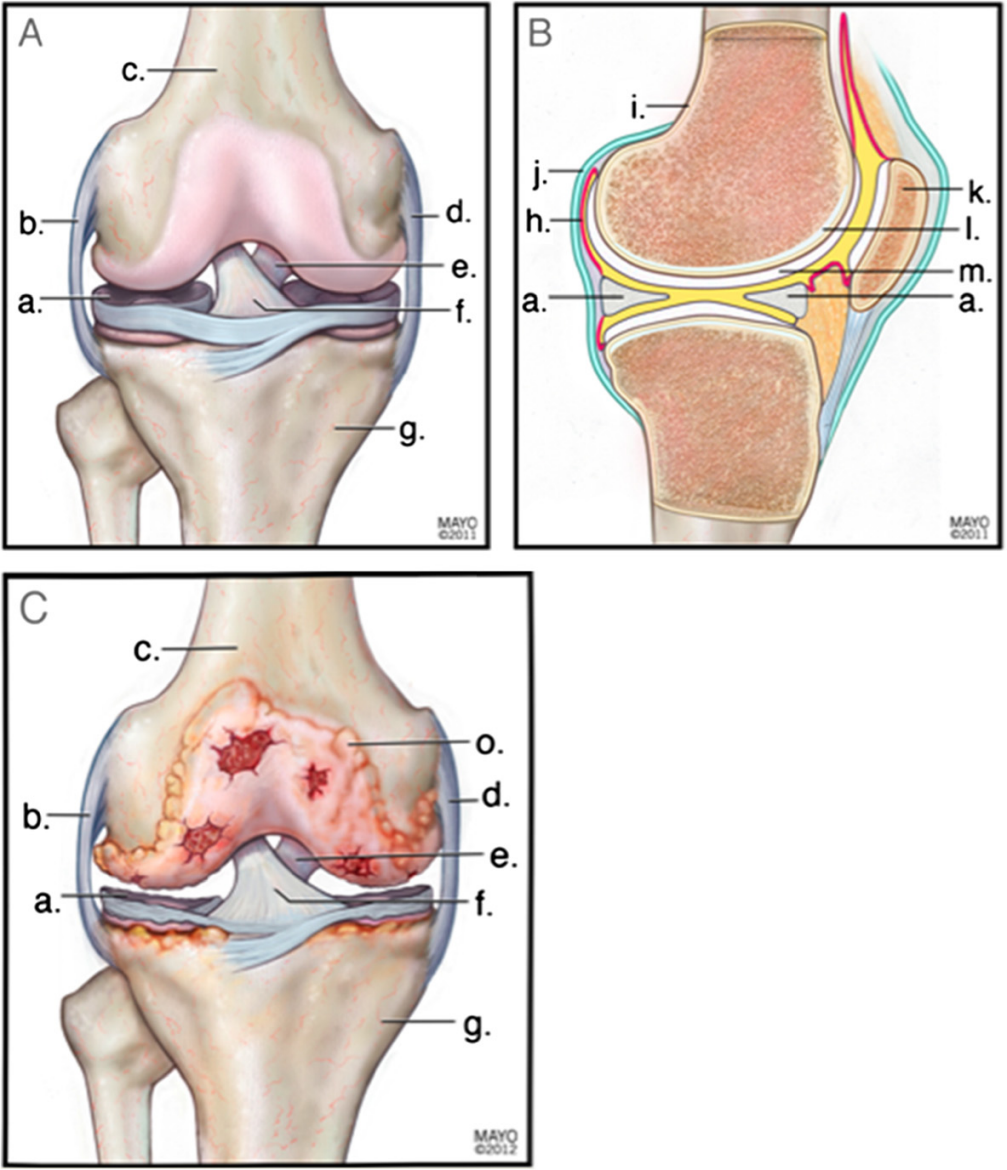
**Anterior (A) and lateral (B) views of a healthy knee joint showing the meniscus (a), lateral collateral ligament (b), distal femur (c), medial collateral ligament (d), posterior cruciate ligament (e), anterior cruciate ligament (f), proximal tibia (g), periosteum (i), joint capsule (j), patella (k), subchondral bone (l), and normal articular cartilage (m).** Degradation of the articular cartilage due to OA is shown in **C**.

Osteoarthritis (OA) is a leading cause of disability in the United States. It is the most common form of arthritis and afflicts 13.9% of U.S. adults aged 25 and older and 33.6% (12.4 million) of those over 65—an estimated 26.9 million individuals in the United States in 2005 [1]. Although knee OA is typically diagnosed from radiograph images showing narrowing of the joint space and osteophytes, all the components of the knee joint are involved [2,3]. Consequently, OA is a progressive disease involving extensive inflammation and damage to not only the joint cartilage and synovium, but also the joint capsule and the bone, muscle, ligaments and tendons surrounding the joint, with alterations in peripheral innervation and central pain processing [4] (Figure 1A,B). The result is irreversible structural change and consequent joint stiffness, pain, and functional impairment [1-4] (Figure 1C).

**Figure 2:**
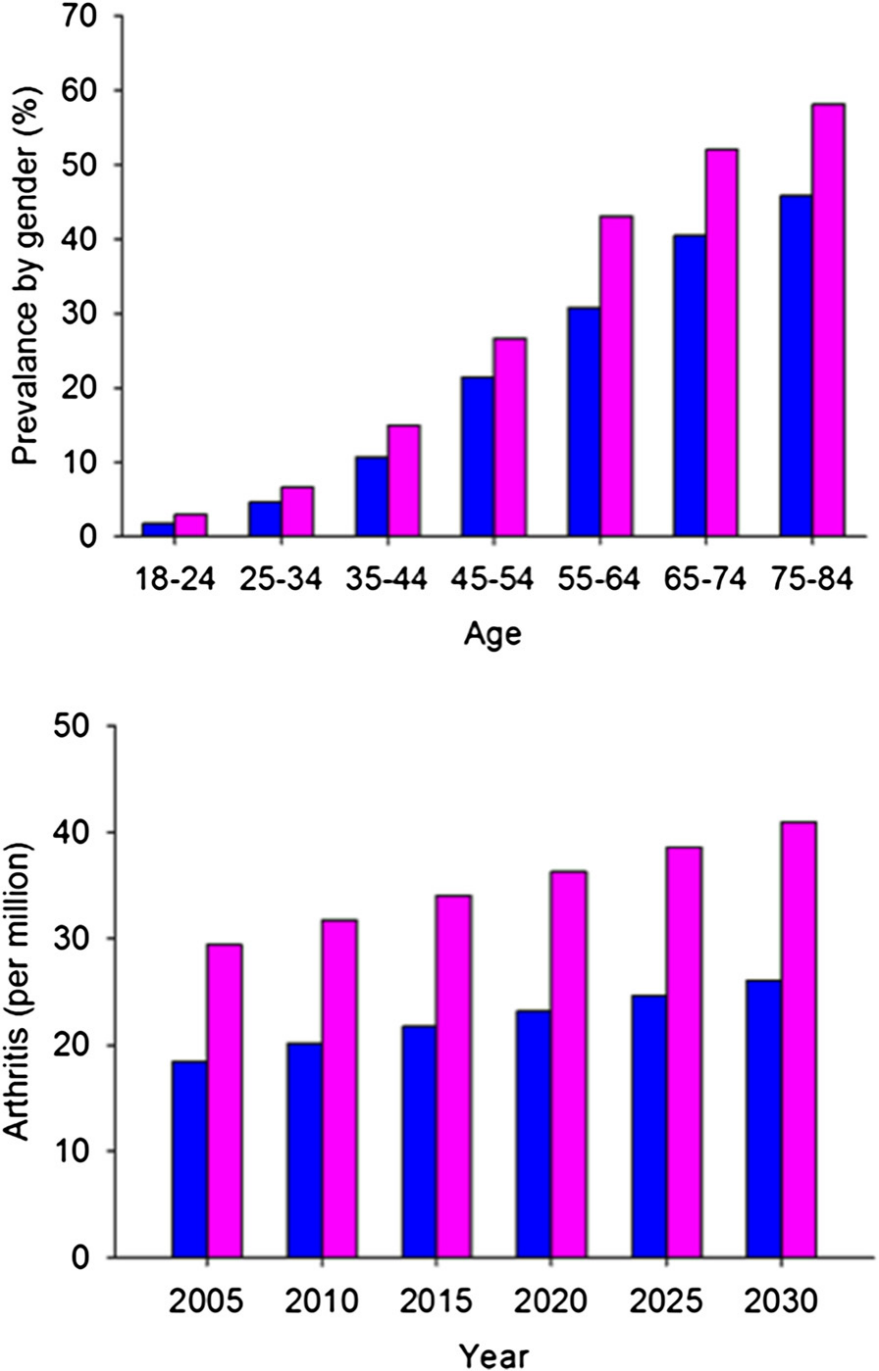
**Prevalence of arthritis by age group for US men (blue) and women (pink) in 2003–2005 (top panel) and current and projected prevalence of arthritis for US men and women (bottom panel).** The graphs are based on data from the Centers for Disease Control website.

Although our understanding of the underlying causes of OA is increasing, we are only beginning to appreciate differences in the disease that exist with respect to sex or gender. Studies sponsored by the Centers for Disease Control and Prevention and the National Institutes of Health have identified differences in the incidence and severity of OA between men and women as well as between racial and ethnic groups [3,5]. The burden of OA is greater in women (Figure 2), who disproportionately develop knee and hand, but not hip OA [5]. The greater number of women in the aging U.S. population is of clinical concern due to the more severe knee OA experienced by women and its impact on quality of life and independence. Moreover, early onset OA is becoming more common, particularly among women who lead physically active lifestyles, such as athletes and workers in occupations that involve exposure to traumatic injury or mechanical stress. Injuries to the anterior cruciate ligament are of particular concern in young women (16–20 years) as approximately 50% of them will develop knee OA within 10–15 years [6,7]. As described in greater detail in the three accompanying papers, these observations underscore the need for research targeted at understanding how sex differences contribute to the development and progression of OA, and influence prevention and treatment strategies.

### Sex differences and models for studying knee OA

Degenerative arthritis has been studied extensively in mice and other laboratory animals such as rats, guinea pigs, rabbits, dogs, and more recently, rhesus monkeys [8]. Such studies have been described as indicative of the human clinical condition because they develop similar histological and morphological abnormalities [8-10] as well as hormonal adaptations and polygenic inheritance in the case of genetic models [11]. Unfortunately, most studies on the mechanisms underlying OA have not considered sex differences, whether the studies used *in vitro* cell culture or animal models.

That sex differences exist in OA was recognized as far back as 1956 [12]. Nonetheless, even with the many animal models that have been developed and studied since that time, few studies have addressed the issue of sex differences. An extensive review of animal models published in 1994 [13] made no mention of sex differences. In 1996, Carlson et al. observed that the prevalence and severity of OA was similar in a limited population of male and female cynomolgus monkeys, whereas knee OA in humans occurs more commonly in females [14]. As recently as 2001 [15], a review of the literature identified the studies had been conducted using sex-matched animals, but these were limited in number and to a small group of laboratories: Silberberg (8 studies, 1941–1963), Walton (6 studies, 1975–1979) and Sokoloff (5 studies, 1956–1962).

The information provided in Table 1, which summarizes all animal studies of osteoarthritis published between 2002 and 2012, underscores the need for well-designed studies addressing sex differences. Out of a total of 1043 studies, only 32 identified the sex of the animals and only one of these made a comparison of the results between the sexes. Clearly, investigators typically do not consider the potential for sex differences in osteoarthritis. Moreover, the Pubmed database listed 2,968 clinical studies of knee OA in the past 10 years with 2,189 (73.7%) specifying the sex of the clinical population. Within this population, only a small percentage identifies "sex" (198 or 9.0%) or "gender" (142 or 6.5%) as a key parameter of the study (Table 2). These statistics indicate that although sex differences are routinely reported in clinical studies, they are not necessarily the principal factor on which the studies are based.

**Table 1:** Summary of osteoarthritis-related journal articles referring to an animal model

**Year**	**Number of OA animal studies**	**Studies indicating sex**	**% indicating sex**	**Number studying sex**
2011	102	2	2	0
2010	156	5	3	0
2009	137	3	2	1
2008	118	3	3	0
2007	110	3	3	0
2006	90	3	3	0
2005	88	7	8	0
2004	91	3	3	0
2003	55	2	4	0
2002	57	1	2	0
2001	39	0	0	0

**Table 2:** List of osteoarthritis-related journal articles specifying sex in an animal model

**Date**	**Journal**	**Authors**	**Animal**	**Sex used**	**Sex studied**
Oct-11	Biochem pharmacol	Imanishi et al.	mice	male and female	No group comparison
Jun-11	J Orthop Res	Watanabe et al.	mouse	male and female	Backcrossing gene
Nov-10	Osteoarthitis Cartilage	Schubert et al.	Wistar rats	male	Experimental vs control
Sep-10	Skeletal Radiol	Liu et al.	New Zealand rabbits	male	Experimental vs control
Aug-10	Ann Rheum Dis	Scott et al.	Dunkin-Hartley guinea pigs	male	Experimental vs control
May-10	Osteoarthritis Cartilage	Gurkan et al.	Hartley guinea pig	male	Experimental vs control
Jan-10	Osteoarthritis Cartilage	Huang et al.	Sprague–Dawley rats	male	Experimental vs control
Apr-09	Int J Exp Pathol	Bowyer et al.	Dunkin-Hartley guinea pigs	male	Experimental vs control
Apr-09	Osteoarthritis Cartilage	Chou et al.	Sprague Dawley rats	male	3 groups
Feb-09	Pain	McDougall et al.	Dunkin-Hartley guinea pigs	male and female	No differences
Sep-08	Osteoarthritis Cartilage	Piscaer et al.	Wistar rats	male	Experimental vs control
Jul-08	Pharm Res	Wang et al.	New Zealand white rabbits	male	Experimental vs control
Jan-08	Am J Physiol Cell	Kitamura et al.	mice	female	Experimental vs control
Jun-07	Arthritis Rheum	Appleton et al.	Sprague Dawley rats	male	Experimental vs control
Jun-07	J Oral Maxillofac Surg	Long et al.	Merino sheep	male	3 time points
May-07	Arthritis Rheum	Wang et al.	New Zealand white rabbits	male	Experimental vs control
Dec-06	J Bone Mineral Res	Kim et al.	piglets	male	Experimental vs control
Aug-06	Arthritis Rheum	Cheung et al.	Hartley guinea pigs, NZW rabbits	male	Experimental vs control
Jun-06	Osteoarthritis Cartilage	Schueiert & MacDougall	Wistar rats	male	Experimental vs control
Nov-05	Arthritis Rheum	Regan et al.	mice	male	2 strains exptl vs control
Sep-05	Osteoarthritis Cartilage	Wadhwa et al.	mice	male and female	Genotyped, not compared
Jul-05	J Orthop Res	Gushue et al.	New Zealand white rabbits	male	Experimental vs control
Apr-05	Arthritis Rheum	Tiraloche et al.	New Zealand white rabbits	male	3 groups
Apr-05	Pain	Pomonis et al.	Sprague Dawley rats	male	Experimental vs control
Feb-05	IEEE Trans Med Imaging	Patel et al.	Wistar Hanover rats	male	Experimental vs control
Feb-05	Osteoarthritis Cartilage	Spriet et al.	New Zealand rabbits	male	Experimental vs control
Dec-04	Acta Orthop Scand	Lahm et al.	Dogs	male	Experimental vs control
Nov-04	Osteoarthritis Cartilage	Papaioannou et al.	New Zealand rabbits	male	Experimental vs control
Sep-04	Am J Sports Med	Murray et al.	cow, sheep, dog, human	Men and women	Animal sex unknown
Nov-03	Toxicol Pathol	Guzman et al.	Wistar rats	male	Experimental vs control
Jun-03	Osteoarthritis Cartilage	Ciombor et al.	Hartley guinea pigs	male	Experimental vs control
Mar-02	Osteoarthritis Cartilage	Muehleman et al.	New Zealand rabbits	Male	4 groups

Cell culture studies are similarly limited with respect to the sex of the cell source. For those studies that use primary cells, whether from animals or humans, most do not directly compare the same set of experimental parameters for both sexes. Moreover, there is often a lack of a statistical design that provides sufficient power to adjust for inter-human variability. When cell lines are used, the sex of the cell is generally not provided. Thus, statements are made concerning underlying mechanisms that may not be accurate for the broader population of subjects.

### The knee as an organ

Diagnosis of knee OA is based on evidence of joint pain or reduced space between articulating bone surfaces due to thinning of the opposing articular cartilages. However, multiple tissues that comprise the knee joint appear to be compromised by the disease, including subchondral bone, articular cartilage, meniscus, anterior cruciate ligament, synovium and synovial fluid, and the innervation of these tissues. A change in any of these tissues can influence the distribution of load across the joint, with corresponding adaptations in the other tissues and ultimately the cartilages [2]. Such pathophysiologic changes may exacerbate age-related physiologic changes in joint function attributable to genetic characteristics, age, sex, and health status, leading to greater cartilage damage. Thus, to better understand how knee OA is differentially expressed in males and females, it is critical to view the knee as an organ, rather than focusing only on the articular cartilage [2,3]. Moreover, the development of OA can involve multiple mechanisms, including mechanical loading, fluctuations in hormonal levels, and modulation of nervous system pathways.

#### Biomechanics and etiology of knee OA

Experimental and computational data suggest that contact stress in joint cartilage is a significant predictor of the risk for developing knee OA. Limb alignment, a major determinant of mechanical stresses within the knee, can predict the development of radiographic signs of knee OA, but these data do not indicate how limb alignment could contribute to sex differences. Similarly, little is known about how sex-related changes in muscle function might contribute to the worsening of knee OA. Significant gaps in knowledge remain as to how changes in musculoskeletal traits, such as obesity, disturb the normal mechanical environment of the knee joint and contribute to sex differences in the initiation and progression of knee OA [16].

#### Hormonal modulation of the knee

Knee tissues are modulated by sex hormones during tissue development and throughout the life cycle in both males and females. Whereas menopause in women is associated with an increase in OA severity, systemic estrogen alone cannot explain the observed sex differences [17]. Recent data, for example, show that sex-specific variations in the responses of chondrocytes to sex steroids are due to differences in receptor number as well as mechanisms of hormone action [18]. Moreover, the reduction in systemic estrogens is accompanied by changes in the relative levels of other steroid hormones, but how this impacts knee physiology is not known.

#### Neurologic contributions to knee OA

In addition to a greater prevalence of knee OA, women also often report greater pain and more substantial reductions in function and quality of life than men [19]. OA pain can be related to the innervation of the knee joint, but the pain does not always match the degree of injury and can continue even after total joint replacement. The mechanisms underlying these differences in pain between men and women with knee OA are unknown [20]. By improving our understanding of the mechanisms responsible for sex differences in the perception of pain in OA, more effective, and possibly sex-specific, treatment strategies will emerge.

## Conclusion

Epidemiologic studies have established that there are sex differences in the incidence and severity of knee OA. Therapeutic approaches to the treatment of OA, particularly regenerative medicine strategies, have not yet taken these sex differences into consideration [21,22]. Effective interventions, however, will require a better understanding of the mechanisms involved in the disease and its differential expression in men and women. Although little is known about the mechanisms that underlie disparities between men and women in disease incidence and severity, it is likely that mechanical, hormonal, and neural events in the joint are involved. The papers that follow in this series review the literature related to sex differences and OA and identify gaps in our understanding with the goal of motivating research on this important problem.

## Competing interests

The authors declare that they have no competing interests.

## Authors’ contributions

This paper was written by the authors to provide an overview of the gaps of knowledge that exist in the study of sex differences related to osteoarthritis. BDB wrote the initial draft, which was then edited by the other authors. All authors read and approved the final manuscript.
